# Beyond Motor Scheme: A Supramodal Distributed Representation in the Action-Observation Network

**DOI:** 10.1371/journal.pone.0058632

**Published:** 2013-03-05

**Authors:** Emiliano Ricciardi, Giacomo Handjaras, Daniela Bonino, Tomaso Vecchi, Luciano Fadiga, Pietro Pietrini

**Affiliations:** 1 Laboratory of Clinical Biochemistry and Molecular Biology, Dept. of Surgery, Medical, Molecular, and Critical Area Pathology, University of Pisa, Pisa, Italy; 2 MRI Lab, Fondazione “G. Monasterio” Regione Toscana/C.N.R., Pisa, Italy; 3 Dept. of Brain and Behavioral Sciences, University of Pavia, Pavia, Italy; 4 Brain Connectivity Center, IRCCS Mondino, Pavia, Italy; 5 Section of Human Physiology, University of Ferrara, Ferrara, Italy; 6 RBCS Department, Italian Institute of Technology, Genova, Italy; 7 Clinical Psychology Branch, Pisa University Hospital, Pisa, Italy; University of Rome, Italy

## Abstract

The representation of actions within the action-observation network is thought to rely on a distributed functional organization. Furthermore, recent findings indicate that the action-observation network encodes not merely the observed motor act, but rather a representation that is independent from a specific sensory modality or sensory experience. In the present study, we wished to determine to what extent this distributed and ‘more abstract’ representation of action is truly supramodal, i.e. shares a common coding across sensory modalities. To this aim, a pattern recognition approach was employed to analyze neural responses in sighted and congenitally blind subjects during visual and/or auditory presentation of hand-made actions. Multivoxel pattern analyses-based classifiers discriminated action from non-action stimuli across sensory conditions (visual and auditory) and experimental groups (blind and sighted). Moreover, these classifiers labeled as ‘action’ the pattern of neural responses evoked during actual motor execution. Interestingly, discriminative information for the action/non action classification was located in a bilateral, but left-prevalent, network that strongly overlaps with brain regions known to form the action-observation network and the human mirror system. The ability to identify action features with a multivoxel pattern analyses-based classifier in both sighted and blind individuals and independently from the sensory modality conveying the stimuli clearly supports the hypothesis of a supramodal, distributed functional representation of actions, mainly within the action-observation network.

## Introduction

The ability to understand others’ actions and intentions from distinct sensory clues is central for daily social interactions. The human mirror system (hMS), as part of a broader action-observation network (AON [1], [2]), plays a major role in this function [3], [4], [5]. The hMS is activated both when individuals perform a goal-directed action and when they observe another individual performing the same action. For this reason, the hMS is considered able to transform sensory information into (motor) knowledge and, through this mechanism, to mediate understanding of motor acts from others [6], [7].

Recent studies have proposed that the representation of actions within the premotor, inferior frontal, parietal and temporal regions of the AON may be based on a distributed and overlapping functional organization [8], [9], similarly to what has already been described for the representation of objects and sounds in other cortical areas (e.g. [10], [11], [12]). Distributed brain responses in specific subregions of the action-responsive fronto-parietal network can be used to discriminate the content, the effectors, or even the behavioral significance of different motor acts, when actions are either observed or performed, or even covertly imagined [8], [9], [13], [14], [15], [16]. Therefore, various subregions within the AON are differentially recruited to control or define specific aspects of motor acts, and the overall response of the AON network seems to contribute to the cross-modal visuo-motor coding of distinct actions [13], [17].

However, it is still unknown whether this distributed representation of actions is shared across sensory modalities. How do we mentally represent ‘hammering’ when just listening to the strikes on a nail, or ‘knocking’ when recognizing the hitting on a door? Though originally both in monkeys and humans the mirror system was thought to rely on visuomotor features, a neural response within the mirror areas has been demonstrated also when simply hearing the sound of an action [18], [19], [20], [21], [22], [23], [24]. Furthermore, individuals that had no previous visual experience still retain the ability to learn actions and behaviors from others. To this purpose, we previously showed that congenitally blind individuals activate a premotor-temporo-parietal cortical network in response to aurally presented actions that overlaps both with hMS areas found in sighted subjects in response to visually and aurally presented stimuli, and with the brain response elicited by motor pantomime of the same actions [25]. Altogether, these findings indicate that the hMS, as part of the AON, codes not merely the observed motor act, but rather a more abstract representation that is independent from a specific sensory modality or experience [17], [26], [27], [28].

Nonetheless, whether the more abstract representation of action is truly *supramodal*, i.e. shares a common coding across sensory modalities, is still unknown. To this aim, we used a pattern recognition approach to analyze neural responses in sighted and congenitally blind individuals during visual and/or auditory perception of a set of hand-made actions, and during the actual motor pantomime of manipulation tasks [25].

Specifically, we used a pattern-classification approach (multi-voxel pattern analysis - MVPA) to decode the information that is represented in a spatially distributed pattern of activity, and to identify as well those brain regions that significantly contribute to the discrimination [29], [30]. We first expected that an MVPA would be able to distinguish between the neural patterns associated with auditory and visual stimuli of actions and non-actions using distributed patterns of response in both sighted and blind individuals. Then, we posited that, because of the hypothesized *supramodal* nature of action representation, an MVPA would be able to classify action and non-action stimuli across the visual and auditory modalities and across the sighted and blind groups, and to recognize as an ‘action’ the neural patterns associated with actual motor performances.

## Materials and Methods

As described in greater details in the original report of the present dataset [25], we used a functional magnetic resonance imaging (fMRI) sparse sampling six-run block design to examine neural activity in congenitally blind and sighted healthy volunteers while they alternated between auditory presentation of hand-executed actions or non-action environmental sounds, and execution of a ‘virtual’ tool or object manipulation task (motor pantomime). In the sighted group, three additional runs were acquired during a visual version of an identical task of motor pantomime and presentation of action or environmental movies.

### Subjects

Eight blind (six female, mean age ± S.D.: 44 ± 16 years) - seven with congenital blindness (causes of blindness: congenital glaucoma, retinopathy of prematurity, and congenital optic nerve atrophy) and one who became completely blind at age 2 years due to congenital glaucoma and had no recollection of any visual experience - and 14 sighted (five female, 32 ± 13 years) right-handed healthy individuals were recruited for the study. All subjects received a medical examination, including routine blood tests and a brain structural MRI scan to exclude any disorder that could affect brain function and metabolism, other than blindness in the blind group.

### Ethics Statement

All participants gave their written informed consent after the study procedures and potential risks had been explained. The study was conducted under a protocol approved by the University of Pisa Ethical Committee (protocol n. 1616/2003), and was developed in accordance with the Protocol of Helsinki (2008).

### Auditory Stimuli

Twenty action and ten environmental sound samples [44.1 Hz, 16 bit quantization, stereo, Free Sound Project, average mean square power and duration normalized] were presented by a MR-compatible pneumatic headphone system (PureSound Audio System Wardray Premise). Speech commands for the motor pantomime task were digitally recorded names of objects/tools to be virtually handled, and a beep sound after 10 s signaled the subject to stop executing the action. Both sounds and speech commands lasted for 10 s.

### Visual Stimuli

Ten second long movies of action and environmental scenes were presented on a rear projection screen viewed through a mirror (visual field: 25° wide and 20° high). Motor commands were triggered by words. Each action showed images of the action being performed by the right hand of an actor viewed from a third person perspective.

### Image Acquisition and Experimental Task

Gradient echo echoplanar (GRE-EPI) images were acquired with a 1.5 Tesla scanner (Signa General Electric). A scan cycle was composed of 5-mm-thick 21 axial slices (FOV = 24 cm, TE = 40 ms, FA = 90, 128×128 pixels) collected in 2,500 ms followed by a silent gap of 2,500 ms (sparse sampling). We obtained six time series of 65 brain volumes while each subject listened to sounds, and three time series while the sighted volunteers only looked at movies. Stimuli were randomly presented with an interstimulus interval of 5 s. Each time series began and ended with 15 s of no stimuli.

During the auditory scanning sessions, volunteers were asked to listen to and recognize sounds while keeping their eyes closed, and also to execute the motor pantomimes when randomly prompted by a human voice command naming a specific tool. During the visual sessions, volunteers were asked to look at movies, and to execute the motor pantomimes when prompted by words. Sensory modality (auditory or visual) was constant for each time series, but auditory and visual runs were alternated in randomized order across sighted subjects. Fifteen stimuli were presented in each time series (equally distributed across stimulus classes) and randomly intermixed with five target pantomime commands. Stimulus presentation was handled by using the software package Presentation® (http://www.neurobs.com). High-resolution T1-weighted spoiled gradient recall images were obtained for each subject to provide detailed brain anatomy.

### MultiVoxel Pattern Analysis

We used the AFNI package (http://afni.nimh.nih.gov/afni - [31] and related software plug-ins for data preprocessing and the BrainVISA/Anatomist package (http://brainvisa.info) for visual rendering of functional imaging data. After standard preprocessing (the different runs were concatenated, co-registered, temporally aligned, and spatially smoothed with an isotropic Gaussian filter, σ = 2.5 mm) [25], the BOLD response magnitude to each stimulus was modeled with a separate regressor in a deconvolution analysis, and calculated by averaging the β-weights of the second, third and fourth volumes of each gamma impulsive response. The decision to use β estimates instead of the less noisy response-amplitude estimate t-values [32] was based on our intention to test classification performances across different stimulus categories whose β and standard error estimates may diverge, and thus result to be more sensitive to differences. The response patterns of each stimulus were transformed into the Talairach and Tournoux Atlas [33], and resampled into isometric 2 mm voxels for group analysis. A template included in the AFNI distribution (the ‘Colin’ brain [34] was used to select cortical voxels.

Furthermore, all the response magnitudes to each stimulus were scaled from −1 to +1, using a hyperbolic tangent, to generate input vectors for the Support Vector Machine (SVM) classifiers [35], [36]. The software ‘SVMlight’ [37] was used to implement the SVM classifiers. Linear SVM classifiers were trained with a small, fixed, data-driven regularization parameter (see [37] for further details), to avoid overfitting the data during the training phase (i.e. soft margin SVM).

Three distinct linear binary classifiers were built in order to separate the patterns of neural response to action and non-action stimuli across the different experimental conditions, that is in blind (sounds only) and sighted (sounds and videos) individuals. Due to the imbalanced numbers of stimuli across the experimental conditions in the sighted group, non-action sounds (n = 10) and videos (n = 11) were randomly upsampled (doubled) to match the size of the action sound set (n = 20), while action videos (n = 23) were randomly downsampled to match the number of action sounds. This choice of combining up-/down-sampling technique with linear SVM has been already considered robust and effective on the predictive performance of learned classifier [38]. Accordingly, the resulting matrix to be classified was made of 560 examples across all sighted individuals (280 non-action stimuli and 280 action stimuli, as resulting from 20 action and non-action stimuli for 14 subjects) for each classifier, and 320 examples (160 non action stimuli and 160 action stimuli, as resulting from 20 stimuli for 8 subjects) across congenitally blind individuals. In addition, we included 280 examples of motor pantomimes from sighted subjects (140 during the auditory sessions, 140 during the visual sessions for 14 subjects) and 80 examples of motor pantomimes from blind subjects (80 during the auditory sessions, for 8 subjects) for testing the capability of our SVM classifiers to identify the motor gestures as ‘actions’, in accordance with the ‘mirror’ rationale of a brain network responding both to action recognition and action real performance. On a general basis, decoding techniques require a high number of examples/stimuli (action and environmental sounds) as compared to the usually high dimensionality of the feature/voxel space [39]. The relative limited number of stimuli in our experiment does not allow here to combine a whole brain approach (taking into account all the distributed information across the cortex) and a single subject decoding. Thus, the whole dataset of examples was used for an across-subjects classification. This procedure strongly relies on commonalities across the individual patterns of responses in accordance with the aims of the analysis to evaluate action/non action representations across the visual and auditory modalities and across the sighted and blind individuals.

In order to select only those voxels strongly related to the discrimination, we built a procedure combining 4-fold nested cross-validation - NCV [29] and a Recursive Feature Elimination algorithm – RFE [40] to recursively prune irrelevant voxels based on their discrimination ability, and to avoid overfitting in model selection [41]. According to the NCV procedure, the stimuli were first divided in four subsets and each fold was tested using one subset after being trained on the other ones. Each RFE iteration of each fold consisted of several steps and generated a specific feature set. Initially, the classifiers were trained with the examples assigned to the fold. The mean of all feature weights of the support vectors was estimated during training. Further, the absolute values of the weight vectors were calculated, and the 2% of the features with the lowest weights were then discarded. A cluster correction with an arbitrary minimum cluster size of 150 voxels (1,200 µL), nearest-neighbor, was performed to remove small, isolated clusters and to reduce the total numbers of iterations. Finally, a discriminative map was obtained by mapping the weights vector onto the Talairach and Tournoux Atlas. This procedure was iterated until all the features/voxels were discarded. For each iteration, an accuracy performance was computed on the testing set of the fold. Then, comparing the accuracies from all folds and RFE iterations, the best feature sets for the three classifiers of the same fold were selected based on their highest mean accuracy [29], [40], [41].

Potential drawbacks of the application of SVM and RFE subsist both in the choice of the number of voxels to be discarded at each iteration, and in the presence of outliers in the data sample that could lead to suboptimal selection of voxels [42], [43]. To mitigate such possibilities, we used a computational expensive RFE algorithm with a relative low number of discarded voxels, and a normalization of data to diminish the role of outliers, respectively. Moreover, this procedure that combine RFE and 4-fold NCV, generated 4 above chance classifiers that rely on different sets of features/voxels across the three experimental conditions (blind - sounds only - and sighted - sounds and videos-). The accuracy of the best fold and the mean accuracy (±S.D.) of all folds were reported. All subsequent analyses, as described below, were indeed limited to the three classifiers (and their features/voxels) of the best fold.

When the three best classifiers were extracted with the RFE algorithm (action vs. non-action stimuli in blind - sounds only - and sighted - sounds and videos - subjects), our classifiers were tested with the motor pantomime examples to confirm the capability to identify the motor execution of virtual gestures as ‘actions’. In addition, to prove the hypothesis of a more abstract representation of action features, an evaluation across stimulus categories and experimental groups was performed.

To examine the degree of overlap in information across the different sensory modalities/groups and to identify those brain areas contributing to the supramodal representation of actions, using the RFE procedure, we built a common ‘supramodal’ SVM classifier using the training data from all stimulus classes of the best fold. Moreover, the discriminative map of this supramodal classifier was employed in a ‘knock-out’ procedure [44]. First, we created a mask defining the discriminative voxels of the supramodal classifier - ‘knock-out’ mask. This ‘knock-out’ voxels were then removed from the three best discriminative maps and the potential changes (reductions) in the accuracy of our three classifiers were determined. Subsequently, restricting our volume of interest to this ‘knock-out’ map only, we built again three distinct linear SVM classifiers to separate action vs. non-action stimuli and to estimate the potential changes (increases) in classification performances related to this set of voxels within and across experimental conditions.

The classifier accuracy values were tested as significantly different from chance with a permutation test (n = 500), randomly changing the labels of examples during the training phase, to avoid biased performance evaluation related to the oversampled non-action stimuli and the different nature of the experimental stimuli (i.e. sounds, videos, and motor pantomime) [29]. Furthermore, differences in within group accuracy estimates of the original SVM classifier when considering the whole discrimination map (Table 1) vs. when excluding the knock-out voxels (Table 2-B) were assessed separately with a non-parametric Wilcoxon signed-rank test [29]. These differences were then aggregated across comparisons with the Fisher’s method [45].

**Table 1:** Accuracy of each SVM classifiers in a within- and across-experimental condition evaluation.

		Sighted		Blind
		SVM classifier trainedon visual stimuli	SVM classifier trained on auditory stimuli	SVM classifier trained on auditory stimuli
*A. Whole brain*				
Sighted	Visual	80.7%***	n.s.	n.s.
	Auditory	n.s.	75.7%***	n.s.
Blind	Auditory	n.s.	n.s.	76.2%***
*B. After excluding the knock-out map*				
Sighted	Visual	77.1%***	n.s.	n.s.
	Auditory	n.s.	74.3%***	n.s.
Blind	Auditory	n.s.	n.s.	73.7%***
*C. By restricting to the knock-out supramodal map*				
Sighted	Visual	73.6%***	61.1%**	n.s.
	Auditory	59.6%*	67.1%***	57.7%*
Blind	Auditory	60.3%**	61.5%**	70.0%***

*** p-value < 0.005,

** p-value < 0.01,

* p-value < 0.05 at permutation test.

**Table 2:** Brain regions obtained with a “knock out” procedure to examine the degree of overlap in information between the representations of different experimental conditions/groups.

Brain areas			Coordinates		
	Hem	BA	x	y	z
Superior Frontal	R	10	5	63	−2
	L	6	−7	−1	64
Middle Frontal	R	6	25	−3	58
Inferior Frontal	R	44	45	9	28
Anterior Cingulate	R	24	5	17	20
Postcentral	L	3	−27	−33	42
	L	3	−35	−31	52
Superior Parietal	L	7	−25	−65	62
Inferior Parietal	R	40	55	−43	28
	L	40	−43	−33	46
Superior Temporal	R	38	41	7	−18
	L	22	−45	−9	−6
Middle Temporal	R	21	55	−35	4
Fusiform	R	19	38	−69	−16
	L	37	−45	−61	−18
Parahippocampal	L	28	−28	−6	−20
Cuneus	R	17	9	−87	6
Middle Occipital	L	19	−39	−73	8

## Results

### MVPA Discrimination of Action and Non-action Stimuli by using Sensory Modality- and Group-specific Classifiers within Condition

In the MVPA, the SVM classifiers that had been trained separately for each group (sighted and blind individuals) and for each sensory modality (auditory and visual) were able to discriminate action from non action stimuli with a accuracy ranging from 75.7% to 80.7% (mean accuracy±S.D. across folders: sighted group, SVM classifier trained on visual stimuli = 76.6±0.04%; SVM classifier trained on auditory stimuli = 72.1±0.03%; blind group, SVM classifier trained on auditory stimuli = 74.4±0.05%) (Table 1-A).

**Figure 1:**
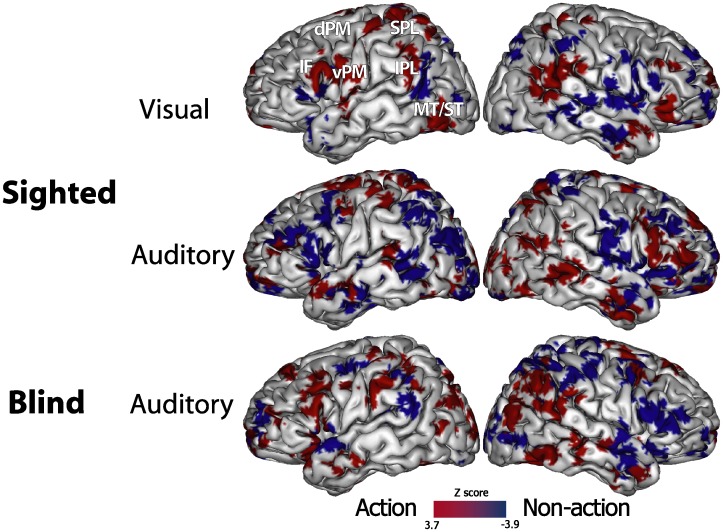
Discriminative maps of the three distinct linear binary SVM classifiers to separate action (red scale) from non-action (blue scale) stimuli in sighted (sounds and videos) and blind (sounds only) subjects, as obtained by using a RFE algorithm. Color intensity reflects the weights of the support vectors, after transformation into Z scores. Spatially normalized volumes are projected a single-subject inflated pial surface template in the Talairach-Tournoux standard space. Ventral and dorsal areas of the premotor cortex (vPM e dPM), inferior frontal (IF) cortex, superior and middle temporal gyri (ST/MT), superior (SPL) and inferior parietal lobule (IPL).

The discrimination maps for each SVM classifier are shown in Figure 1. Middle and inferior frontal, premotor, inferior and superior parietal and middle/superior temporal regions, predominantly in the left hemisphere, provided the most relevant information for stimuli classification. Furthermore, specific differences in the discrimination maps were visible among different experimental conditions, and additional discriminative voxels were found in bilateral striate and extrastriate regions, dorsolateral and medial prefrontal cortex, anterior cingulate, and precuneus.

### MVPA Discrimination of Action and Non-action Stimuli by using Sensory Modality- and Group-specific Classifiers Across Conditions and Groups

The across-condition and across-group evaluation showed that the SVM-classifier trained within condition did not reach a significant discrimination accuracy between action vs. environmental stimuli (Table 1-A).

### MVPA Discrimination of Action and Non-action Stimuli by using a Combined ‘Supramodal’ Classifier and the “Knock out” Approach

To test the more abstract representation of action feature, we defined a combined supramodal classifier and used a “knock out” procedure to examine the degree of overlap in information between the representations of actions across the different experimental conditions and groups.

**Figure 2:**
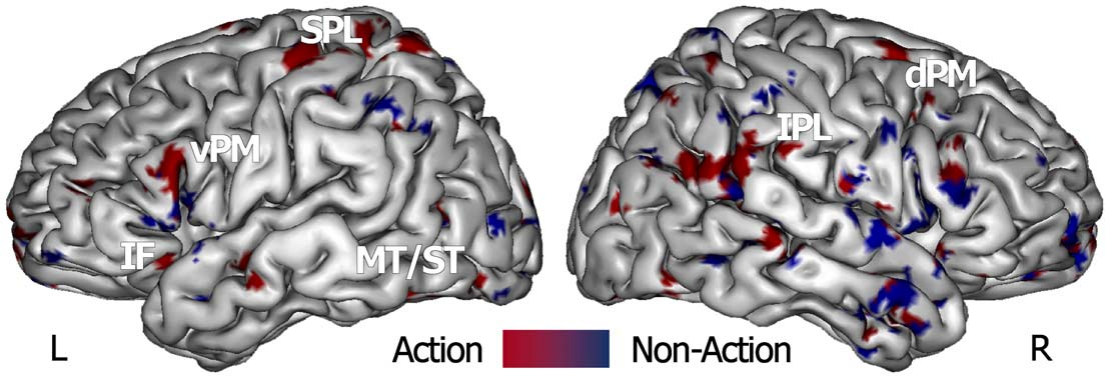
Map of the combined ‘supramodal’ SVM classifier that was defined by using the training data from all action (red scale) and non-action (blue scale) stimuli classes, and was employed in a ‘knock-out’ procedure. Spatially normalized volumes are projected onto a single-subject inflated pial surface template in the Talairach-Tournoux standard space. Ventral and dorsal areas of the premotor cortex (vPM e dPM), inferior frontal (IF) cortex, superior and middle temporal gyri (ST/MT), superior (SPL) and inferior parietal lobule (IPL).

The combined supramodal classifier was able to recognize the action feature with an overall accuracy of 66.7% (p-value < 0.005 at permutation test). Within its discriminative map, we identified voxels that were mainly located in AON areas [2], such as the left superior parietal, right inferior parietal, bilateral ventral and right dorsal premotor area, bilateral middle/superior temporal cortex (Figure 2, Table 2). Additional common voxels were found in bilateral striate and extrastriate, dorsolateral and medial prefrontal cortex, anterior cingulate, bilateral precuneus and posterior cingulate cortex.

The “knock out” procedure, that is, the exclusion of the discriminative areas defined by the supramodal classifier from the three best discriminative maps, resulted in a significantly decreased accuracy as compared to the original within-category discriminations for the SVM classifiers in both sighted and blind individuals (p<0.05, Wilcoxon signed-rank test and Fisher’s method), though they were still able to perform a significant within-category discrimination (Table 1-B). Still, no across condition/group discrimination resulted significant (Table 1-B).

In contrast, when relying just on these supramodal voxels, all three SVM classifiers were able to reach a significant within-category discrimination, and the across experimental condition/group discriminations showed significant accuracies (Table 1-C). In details, the visual SVM classifier showed significant accuracy in classifying auditory stimuli sessions in sighted and blind subjects. Also the auditory SVM classifiers trained in sighted and blind individuals correctly performed an action vs. non action discrimination across stimuli categories (i.e. sensory modality) and experimental groups, with the only exception of visual stimuli for the auditory SVM classifier trained in blind individuals (Table 1-C).

### Discrimination of Motor Pantomimes

As detailed in Table 3-A, in a whole brain approach, the SVM classifier trained on visual stimuli for the sighted group was significantly able to recognize as ‘actions’ the motor pantomimes in sighted individuals with a high accuracy of discrimination (85.3%). At a more conservative level, none of the auditory SVM classifiers as trained in either sighted or blind individuals were able to recognize as ‘actions’ the motor pantomimes in sighted individuals; in addition, none of the classifiers were able to significantly recognize as ‘actions’ the neural patterns of motor pantomime in the blind individuals. In addition, the neural responses during motor pantomime performance were still recognized as ‘actions’ by both the visual and the auditory SVM classifiers of sighted individuals when limiting to the discrimination map of the supramodal classifier (Table 3-B), further indicating that these functional overlapping voxels mainly contribute to the representation of motor acts. When volume of interest was restricted to the discrimination map of the supramodal classifier (Table 3-C), only the SVM classifier trained on the visual stimuli in sighted individual was significantly able to classify the neural responses during motor pantomime performances in sighted individuals.

**Table 3:** Accuracy of each SVM classifiers in recognizing motor pantomime as ‘action’.

	Sighted		Blind
	SVM classifier trained on visual stimuli	SVM classifier trained on auditory stimuli	SVM classifier trained on auditory stimuli
*A. Whole brain discrimination*			
Sighted	85.3%***	73.9%*	53.2%*
Blind	60%*	65%*	61.2%*
*B. After excluding the knock-out map*			
	84.3%***	71.7%*	46.4%
	57.5%*	63.7%*	65%*
*C. By restricting to the knock-out map*			
Sighted	85.3%***	76.8%**	67.5%*
Blind	70%*	52.5%	61.5%*

a visual and auditory runs have been considered together.

*** p-value < 0.005,

** p-value < 0.05 at permutation test;

* p-value < 0.05 at the binomial test.

On the other hand, when using a less conservative approach with a binomial test, both SVM classifiers trained on auditory stimuli of sighted and blind individuals were able to recognize as ‘actions’ the motor pantomimes in a whole brain discrimination (Table 3-A), worsened their accuracy performance after excluding the knock-out map (Table 3-B), and improved when limiting to the discrimination map of the supramodal classifier (Table 3-C).

Finally, the combined supramodal classifier was able to recognize as ‘actions’ the motor pantomimes both on visual stimuli (accuracy 85.3%, p-value < 0.005 at permutation test) and on auditory stimuli (accuracy 76.8%, p-value < 0.05 at permutation test) in sighted individuals.

## Discussion

Here we used a multivariate pattern recognition method to distinguish neural responses in congenitally blind and sighted participants during the visual and auditory perception of a set of hand-made action and environmental stimuli, to test the hypothesis that motor acts are represented in a distributed and truly *supramodal* fashion. In a ‘classical’ univariate analysis of the same functional dataset, we had previously shown that blind individuals activate a premotor-temporo-parietal network that subserves a ‘mirror’ response to aurally presented actions, and that such a network overlaps with the hMS, as part of the AON, found in sighted individuals in response to both visually and aurally presented stimuli [25]. However, we did not assessed whether this ‘more abstract’ representation of action is truly *supramodal*, i.e. shares a common coding across visual and auditory sensory modalities. Recently, MVPA has been employed to study the representation of different categories of stimuli within the same perceptual modality [29], [30]. In this study, for the first time, an MVPA specifically evaluated the representation of the same stimulus category (action) within and across different sensory modalities (visual, auditory or motor) and experimental groups (sighted and congenitally blind).

### Action Discrimination by using Sensory Modality- and Group-specific Classifiers within Condition

Single MVPA-based classifiers, trained separately for each experimental condition and group, were able to significantly discriminate action from non action stimuli within condition. These observations in sighted and congenitally blind individuals using visual and auditory stimuli showed for the first time that sounds can be successfully used to distinguish neural responses to action as compared to environmental stimuli, and expand previous functional studies that explored the distributed and overlapping representation of visually-presented motor acts using MVPA-based approaches [8], [9], [13], [14], [15], [16].

As a matter of facts, distributed activity in left and right anterior intraparietal cortex has been previously used to discriminate the content of three different motor acts (‘*rock-paper-scissors*’ game), when these actions were either observed or performed [9]. Action-dedicated regions were also described in lateral occipito-temporal and left postcentral/anterior parietal cortex: the postcentral area carries distributed information about the effectors used to perform the action, while parietal regions about the action goal [8]. Similarly, while viewing videos of different motor acts, distributed action representations can be clustered according to the specific relationship between agent and object (i.e. their behavioral significance) in the inferior parietal cortex, or according to the effector (foot, hand, mouth) used to perform the action in the premotor cortex [13], although both effector-dependent and effector independent representations have been shown coexist in this inferior frontal and precentral region [46].

In our study, the discrimination maps of the three classifiers (Figure 1) identified the most relevant information about ‘action feature’ as primarily located in a distributed and bilateral, though left prevalent, prefrontal, premotor, parietal and temporal circuit. This network of discrimination of action from non action stimuli mainly included brain regions within the hMS-related action recognition network [2], as the inferior and superior parietal, intraparietal, dorsal and ventral premotor, and inferior frontal areas.

Nonetheless, specific differences across sensory conditions and groups also were present. For instance, in sighted subjects the classifier trained on auditory stimuli relied more on right inferior frontal regions while homologous left areas contributed more to visual action recognition. This may suggest that the features that can be extracted by MVPA may not be directly linked to action *per se*, but rather be related to other physical/semantic attributes of the stimuli (e.g. dynamicity, imageability, conveying modality, onset time, etc. – for a detailed list of stimuli, refer to [25] or to their specific contents (presence/absence of manipulable objects, presence/absence of living/non living entities, etc.), and thus be processed across different brain areas [29], [30], [47]. Furthermore, we cannot exclude that the discriminative maps for each experimental condition might have been influenced by stimulus-specific differences in the switch from resting activity to task-associated response, due to a different Default-Mode Network (DMN) recruitment by the two stimulus classes. Nonetheless, when observing brain regions involved in the discrimination of stimuli, no specific DMN regions appear to consistently contribute to action vs. environmental stimulus classification. In addition, the current experimental setup could hardly induce DMN-related self-oriented or introspective activities.

Each action was carefully chosen to be as different from the others as possible, and distinct sets of actions were selected for the visual, auditory and motor pantomime conditions. In addition, our subsequent analyses aimed at determining action coding across different sensory modalities (visual, auditory or motor) and experimental groups, thus enhancing the common features of action representation and limiting any possible confounds related to stimuli selection. Even so, a protocol for a MVPA approach would have benefit from better-selected control or baseline conditions, and balanced/matched stimuli across classes to modulate for a wider gamut of features.

### Action Discrimination by using Both Sensory Modality- and Group-specific Classifiers Across Conditions/groups and a Combined ‘Supramodal’ Classifier

To what extent are the distributed representations of actions related to the specific sensory modality used for action perception?

First, to validate the degree of overlap in information across the experimental conditions (i.e., visual or auditory modality) and groups, the classifiers that had been trained on each single experimental condition were employed in an across sensory modalities and groups evaluation. The three classifiers (i.e., visual stimuli in sighted individuals, auditory stimuli in sighted and in blind subjects) were unable to discriminate the action features across conditions or groups. This confirms our previous consideration that specific differences in the features that are ‘employed’ by the single classifiers across sensory conditions and groups were present.

Second, the same pattern recognition approach was applied across experimental conditions to define a combined classifier. Indeed, accordingly to our hypothesis of a more abstract representation of actions, this classifier was able to discriminate significantly action stimuli, independently from both the sensory modality conveying the information and the experimental group. The brain areas belonging to the AON - specifically superior and inferior parietal, ventral and right dorsal premotor, middle/superior temporal areas - primarily contributed to this discrimination ability.

Third, this hMS-based supramodal network was then utilized in a ‘knock-out’ procedure [44]. We reasoned that if these common voxels of the combined classifier retain a more abstract functional representation of actions, then their subtraction from the distinct linear binary classifiers should results in a significantly diminished accuracy in separating action vs. non-action stimuli. Conversely, if this network retains a reliable representation of actions, when limiting only to the supramodal common areas, the distinct classifiers should both maintain significant discrimination accuracies within experimental conditions (comparable with the accuracy levels of the whole brain classification), and have a greater accuracy to recognize action stimuli across experimental conditions.

As expected, the removal of functional overlapping voxels significantly decreased the within-condition discrimination accuracy of the classifiers that had been trained on each single condition, as compared to the original classification performance. On the other hand, when extracting the common representation of actions, the classifiers both maintained a significant within-condition discrimination accuracy and improved accuracies across-conditions. Both the classifiers trained on visual and auditory stimuli in sighted individuals and the classifier trained in blind individuals showed a significant across conditions/groups discrimination of action vs. non action stimuli. Once more, the value of the combined classifier and the “knock-out” procedure in enhancing the characterization of the supramodal representation of actions relies on the possibility to identify those common brain regions that contribute to discriminate action from non-action perception across the different experimental conditions, independently from the specific features that could drive stimuli separation within a single sensory modality.

### Motor Pantomimes Distinguished as Actions

In line with the ‘mirror rationale’ that the distributed and supramodal representation of actions should retain substantial information during both action recognition and performance, we also tested whether patterns of neural response for actual motor performance were recognized as ‘actions’ from a specific MVPA-based classifiers. Since our test set was unbalanced, as the SVM classifiers were used to recognize ‘actions’ in motor pantomimes but with no alternative control conditions, we applied a more conservative correction, in order to limit false positive results.

In a whole brain approach, only the visual classifiers of sighted participants was significantly able to recognize as an ‘action’ the patterns of neural response to motor pantomimes in sighted individuals but not in the blind ones. Interestingly, this greater discrimination ability of the classifier trained on visual stimuli in sighted individuals across the different analyses may be related to the somehow prevalent visuomotor nature of the hMS itself, as shown by the stronger and more extended unimodal visual response of this action-specific network (Gazzola et al., 2006; Ricciardi et al., 2009). This discrimination ability became slightly smaller when excluding the ‘knock-out’ supramodal map, and, conversely, remained equally accurate when the classification was restricted to the ‘knock-out’ supramodal map. In addition, when restricting to the supramodal map also the auditory classifiers from sighted participants were significantly able to recognize motor acts.

In addition, when using a less conservative approach, both SVM classifiers trained on auditory stimuli of sighted and blind individuals were also able to recognize as ‘actions’ the motor pantomimes. In line with the observations with the visual classifiers of sighted participants, this discrimination ability became smaller or greater when excluding or restricting to, respectively, the ‘knock-out’ supramodal map.

Interestingly, the classification accuracies of the three SVM classifiers trained on either visual or auditory stimuli in sighted and blind individuals resulted the highest when restricted to the ‘knock-out’ supramodal map, thus further supporting a more abstract functional representation of motor acts in these regions belonging to the AON. Consistently, motor pantomimes were correctly classified by the combined supramodal classifier for visual and auditory stimuli in sighted individuals.

### Conclusions

The present study demonstrates for the first time that a MVPA can be used successfully to discriminate functional representations (of actions) in both sighted and blind individuals. The ability to identify (action) features across sensory modalities and experimental groups supports the hypothesis of a distributed and truly supramodal functional representation of actions within the brain areas of the hMS, and leads to two main considerations.

First, these results are consistent with previous functional studies in both sighted and congenitally blind individuals that have shown the existence of supramodal networks able to process external information regardless of the sensory modality through which the information is acquired [12], [27], [28]. Homologies do not only limit to the spatial localization of the patterns of neural activations, but mainly involve the content (i.e. action or non action) of the neural responses: for instance, overlapping category-related patterns of response across sensory modalities have been found in both sighted and congenitally blind individuals (Mahon et al., 2009; Pietrini et al., 2004b). Applied to the assessment of supramodal functional organization, pattern recognition approaches have been employed here to classify neural responses across experimental samples (i.e., congenitally blind and sighted individuals) and sensory modalities, and consequently to localize those cortical regions that functionally contribute to a supramodal representation.

Second, this more abstract functional organization enables congenitally blind individuals to acquire knowledge about different perceptual, cognitive and affective aspects of an external world that they have never seen [12], [27], [28]. The demonstration of a more abstract, sensory independent representation of actions within the hMS supports the rationale of a cognitive system that might play a major role not only in action recognition and intention understanding, but also in learning by imitation, empathy, and language development [7], [17].

## References

[1] Kilner JM (2011) More than one pathway to action understanding. Trends Cogn Sci 15: 352–357. 21775191 10.1016/j.tics.2011.06.005 PMC3389781

[2] Molenberghs P, Cunnington R, Mattingley JB (2012) Brain regions with mirror properties: a meta-analysis of 125 human fMRI studies. Neurosci Biobehav Rev 36: 341–349. 21782846 10.1016/j.neubiorev.2011.07.004

[3] Fadiga L, Fogassi L, Pavesi G, Rizzolatti G (1995) Motor facilitation during action observation: a magnetic stimulation study. J Neurophysiol 73: 2608–2611. 7666169 10.1152/jn.1995.73.6.2608

[4] Rizzolatti G, Fadiga L, Gallese V, Fogassi L (1996) Premotor cortex and the recognition of motor actions. Brain Res Cogn Brain Res 3: 131–141. 8713554 10.1016/0926-6410(95)00038-0

[5] Rizzolatti G, Craighero L (2004) The mirror-neuron system. Annu Rev Neurosci 27: 169–192. 15217330 10.1146/annurev.neuro.27.070203.144230

[6] Fabbri-Destro M, Rizzolatti G (2008) Mirror neurons and mirror systems in monkeys and humans. Physiology (Bethesda) 23: 171–179. 18556470 10.1152/physiol.00004.2008

[7] Gallese V, Keysers C, Rizzolatti G (2004) A unifying view of the basis of social cognition. Trends Cogn Sci 8: 396–403. 15350240 10.1016/j.tics.2004.07.002

[8] Oosterhof NN, Wiggett AJ, Diedrichsen J, Tipper SP, Downing PE (2010) Surface-based information mapping reveals crossmodal vision-action representations in human parietal and occipitotemporal cortex. J Neurophysiol 104: 1077–1089. 20538772 10.1152/jn.00326.2010 PMC2934920

[9] Dinstein I, Gardner JL, Jazayeri M, Heeger DJ (2008) Executed and observed movements have different distributed representations in human aIPS. J Neurosci 28: 11231–11239. 18971465 10.1523/JNEUROSCI.3585-08.2008 PMC2666623

[10] Haxby JV, Gobbini MI, Furey ML, Ishai A, Schouten JL, et al (2001) Distributed and overlapping representations of faces and objects in ventral temporal cortex. Science 293: 2425–2430. 11577229 10.1126/science.1063736

[11] Staeren N, Renvall H, De Martino F, Goebel R, Formisano E (2009) Sound categories are represented as distributed patterns in the human auditory cortex. Curr Biol 19: 498–502. 19268594 10.1016/j.cub.2009.01.066

[12] Pietrini P, Furey ML, Ricciardi E, Gobbini MI, Wu WH, et al (2004) Beyond sensory images: Object-based representation in the human ventral pathway. Proc Natl Acad Sci U S A 101: 5658–5663. 15064396 10.1073/pnas.0400707101 PMC397466

[13] Jastorff J, Begliomini C, Fabbri-Destro M, Rizzolatti G, Orban GA (2010) Coding observed motor acts: different organizational principles in the parietal and premotor cortex of humans. J Neurophysiol 104: 128–140. 20445039 10.1152/jn.00254.2010

[14] Oosterhof NN, Tipper SP, Downing PE (2012) Visuo-motor imagery of specific manual actions: A multi-variate pattern analysis fMRI study. Neuroimage 63: 262–271. 22766163 10.1016/j.neuroimage.2012.06.045

[15] Oosterhof NN, Tipper SP, Downing PE (2012) Viewpoint (in)dependence of action representations: an MVPA study. J Cogn Neurosci 24: 975–989. 22264198 10.1162/jocn_a_00195

[16] Molenberghs P, Hayward L, Mattingley JB, Cunnington R (2012) Activation patterns during action observation are modulated by context in mirror system areas. Neuroimage 59: 608–615. 21840404 10.1016/j.neuroimage.2011.07.080

[17] Cattaneo L, Rizzolatti G (2009) The mirror neuron system. Arch Neurol 66: 557–560. 19433654 10.1001/archneurol.2009.41

[18] Etzel JA, Gazzola V, Keysers C (2008) Testing simulation theory with cross-modal multivariate classification of fMRI data. PLoS ONE 3: e3690. 18997869 10.1371/journal.pone.0003690 PMC2577733

[19] Galati G, Committeri G, Spitoni G, Aprile T, Di Russo F, et al (2008) A selective representation of the meaning of actions in the auditory mirror system. Neuroimage 40: 1274–1286. 18276163 10.1016/j.neuroimage.2007.12.044

[20] Gazzola V, Aziz-Zadeh L, Keysers C (2006) Empathy and the somatotopic auditory mirror system in humans. Curr Biol 16: 1824–1829. 16979560 10.1016/j.cub.2006.07.072

[21] Keysers C, Kohler E, Umilta MA, Nanetti L, Fogassi L, et al (2003) Audiovisual mirror neurons and action recognition. Exp Brain Res 153: 628–636. 12937876 10.1007/s00221-003-1603-5

[22] Kohler E, Keysers C, Umilta MA, Fogassi L, Gallese V, et al (2002) Hearing sounds, understanding actions: action representation in mirror neurons. Science 297: 846–848. 12161656 10.1126/science.1070311

[23] Lewis JW, Brefczynski JA, Phinney RE, Janik JJ, DeYoe EA (2005) Distinct cortical pathways for processing tool versus animal sounds. J Neurosci 25: 5148–5158. 15917455 10.1523/JNEUROSCI.0419-05.2005 PMC6724809

[24] Lewis JW, Frum C, Brefczynski-Lewis JA, Talkington WJ, Walker NA, et al (2011) Cortical network differences in the sighted versus early blind for recognition of human-produced action sounds. Human Brain Mapping 32: 2241–2255. 21305666 10.1002/hbm.21185 PMC3517890

[25] Ricciardi E, Bonino D, Sani L, Vecchi T, Guazzelli M, et al (2009) Do we really need vision? How blind people "see" the actions of others. J Neurosci 29: 9719–9724. 19657025 10.1523/JNEUROSCI.0274-09.2009 PMC6666597

[26] Cattaneo Z, Vecchi T, Cornoldi C, Mammarella I, Bonino D, et al (2008) Imagery and spatial processes in blindness and visual impairment. Neurosci Biobehav Rev 32: 1346–1360. 18571726 10.1016/j.neubiorev.2008.05.002

[27] Ricciardi E, Pietrini P (2011) New light from the dark: what blindness can teach us about brain function. Curr Opin Neurol 24: 357–363. 21677583 10.1097/WCO.0b013e328348bdbf

[28] Kupers R, Pietrini P, Ricciardi E, Ptito M (2011) The nature of consciousness in the visually deprived brain. Frontiers in Psychology 2: 1–14. 21713178 10.3389/fpsyg.2011.00019 PMC3111253

[29] Pereira F, Mitchell T, Botvinick M (2009) Machine learning classifiers and fMRI: a tutorial overview. Neuroimage 45: S199–209. 19070668 10.1016/j.neuroimage.2008.11.007 PMC2892746

[30] Poldrack RA, Halchenko YO, Hanson SJ (2009) Decoding the large-scale structure of brain function by classifying mental States across individuals. Psychol Sci 20: 1364–1372. 19883493 10.1111/j.1467-9280.2009.02460.x PMC2935493

[31] Cox RW (1996) AFNI: software for analysis and visualization of functional magnetic resonance neuroimages. Comput Biomed Res 29: 162–173. 8812068 10.1006/cbmr.1996.0014

[32] Misaki M, Kim Y, Bandettini PA, Kriegeskorte N (2010) Comparison of multivariate classifiers and response normalizations for pattern-information fMRI. Neuroimage 53: 103–118. 20580933 10.1016/j.neuroimage.2010.05.051 PMC2914143

[33] Tailairach J, Tournoux P (1988) Co-Planar Stereotaxic Atlas of the Human Brain. New York: Thieme Medical Publisher, Inc.

[34] Holmes CJ, Hoge R, Collins L, Woods R, Toga AW, et al (1998) Enhancement of MR images using registration for signal averaging. J Comput Assist Tomogr 22: 324–333. 9530404 10.1097/00004728-199803000-00032

[35] Schölkopf B, Burges CJC, Smola AJ (1999) Advances in kernel methods: support vector learning. Cambridge, Mass.: MIT Press.

[36] Cortes C, Vapnik V (1995) Support-vector networks. Machine Learning 20: 273–297.

[37] Joachims T (1999) Making large-Scale SVM Learning Practical. In: Schölkopf B, Burges C, Smola A, editors. Advances in Kernel Methods - Support Vector Learning. Cambridge, MA, USA: MIT Press. 41–56.

[38] Japkowicz N, Stephen S (2002) The class Imbalance Problem: A Systematic Study. Intelligent Data Analysis 6: 429–450.

[39] Mitchell T, Hutchinson R, Niculescu R, Pereira F, Wang X, et al (2004) Learning to Decode Cognitive States from Brain Images. Mach Learn 57: 145–175.

[40] De Martino F, Valente G, Staeren N, Ashburner J, Goebel R, et al (2008) Combining multivariate voxel selection and support vector machines for mapping and classification of fMRI spatial patterns. Neuroimage 43: 44–58. 18672070 10.1016/j.neuroimage.2008.06.037

[41] Cawley GC, Talbot NLC (2010) On Over-fitting in Model Selection and Subsequent Selection Bias in Performance Evaluation. Journal of Machine Learning Research 11: 2079–2107.

[42] Guyon I, Elisseef A (2003) An introduction to variable and feature selection. Journal of Machine Learning Research 3: 1157–1182.

[43] Guyon I, Weston J, Barnhill S, Vapnik V (2002) Gene selection for cancer classification using support vector machines. Machine Learning 46: 389–422.

[44] Carlson TA, Schrater P, He S (2003) Patterns of activity in the categorical representations of objects. J Cogn Neurosci 15: 704–717. 12965044 10.1162/089892903322307429

[45] Lowry R (1999) 1999–2007. Concepts and Applications of Inferential Statistics. Available: http://vassarstats.net/textbook/. Accessed 2013 Feb 8.

[46] Cattaneo L, Sandrini M, Schwarzbach J (2010) State-dependent TMS reveals a hierarchical representation of observed acts in the temporal, parietal, and premotor cortices. Cereb Cortex 20: 2252–2258. 20051360 10.1093/cercor/bhp291

[47] O'Toole AJ, Jiang F, Abdi H, Penard N, Dunlop JP, et al (2007) Theoretical, statistical, and practical perspectives on pattern-based classification approaches to the analysis of functional neuroimaging data. J Cogn Neurosci 19: 1735–1752. 17958478 10.1162/jocn.2007.19.11.1735

